# SAFE_DTx: Safety-First Framework for AI-Driven Personalization in Digital Therapeutics

**DOI:** 10.2196/78202

**Published:** 2026-09-25

**Authors:** Dohyoung Rim

**Affiliations:** 1 Rowan Corporation Seoul Republic of Korea

**Keywords:** digital therapeutics, artificial intelligence, AI, patient safety, clinical decision support, personalized medicine, digital health

## Abstract

Digital therapeutics (DTx) are emerging as evidence-based software interventions, but current AI-driven personalization approaches lack dedicated safety-focused frameworks and face challenges due to scarce long-term outcome data and unpredictable model behaviors. We propose SAFE_DTx, a safety-first architectural framework for DTx that integrates well-established principles of predictive modeling and constrained decision-making to prioritize patient safety. SAFE_DTx’s 2-module architecture comprises an AI feedback prediction module that forecasts short-term patient responses and a constrained planning module that selects the next intervention under explicit safety constraints. By decoupling these components and enforcing clear safety guardrails, the framework enables dynamic, real-time adaptation to individual patient feedback while staying within evidence-based safety limits. This modular design also enhances transparency in the decision-making process, and an in silico evaluation demonstrates its preliminary architectural feasibility, showing greater engagement and no safety violations compared to baseline strategies within the simulated environment. SAFE_DTx’s safety-by-design architecture aligns with emerging regulatory emphasis on AI transparency and patient safety. It directly addresses key clinical challenges in AI-driven DTx personalization by ensuring that tailored interventions do not compromise patient safety.

## Introduction

Digital therapeutics (DTx) represent a paradigm shift in health care delivery, offering evidence-based software interventions for conditions ranging from attention-deficit/hyperactivity disorder (ADHD) to chronic pain. However, implementing AI-driven personalization in DTx presents unique clinical challenges that demand careful consideration of patient safety.

Current personalization approaches suffer from 2 critical limitations. First, robust long-term outcome data remain scarce in many DTx domains [1]. This makes it impractical—and potentially unsafe—to directly optimize for long-term outcomes, as decisions would otherwise be made with insufficient feedback and could lead to inappropriate interventions. Second, complex AI models may exhibit unpredictable behavior that could compromise patient safety [2]. Because of these safety concerns, fully autonomous DTx are generally not endorsed in current practice; regulators and clinicians instead prefer AI-driven therapies that include human oversight.

To address these challenges, we propose SAFE_DTx, a safe optimization framework for DTx that shifts the focus to intermediate feedback signals as proximal objectives, rather than final clinical outcomes alone. In SAFE_DTx, an AI module predicts the patient’s likely short-term feedback response (eg, next-day engagement level or symptom change) to each of various therapy options. A separate planner then selects the optimal action based on these predictions while enforcing explicit medical safety constraints (eg, hard limits on intervention intensity or rules for clinical escalation). By optimizing these intermediate feedback signals, the DTx can intelligently adapt to the patient in fine-grained steps while staying within established safety limits. In contrast to existing DTx personalization methods that target distant outcomes and conventional safe reinforcement learning (RL) frameworks that focus purely on algorithmic bounds, SAFE_DTx does not claim algorithmic novelty in its underlying learning mechanisms. Rather, its distinct contribution is an architectural synthesis that uniquely operationalizes these adaptive principles for clinical DTx by structurally separating clinical surrogate prediction from predefined safety constraint filtering. This 2-step strategy draws on principles of model-driven decision support: the AI provides a model of the patient’s near-term dynamics, and the planner computes treatment decisions that maximize cumulative intermediate feedback signals under explicit safety constraints. Although the individual components of SAFE_DTx—such as surrogate outcomes, prediction-planning separation, and constrained optimization—are well established in operations research and safe RL, our specific contribution lies in their unique architectural combination and framing tailored to enforce safety boundaries in DTx.

## Related Work

### AI Personalization in Digital Health

There is a growing body of work applying machine learning, especially RL, to personalize health interventions. In mobile and digital health, RL has been explored to optimize just-in-time adaptive interventions (JITAIs) that deliver support (eg, motivational messages or therapy prompts) at moments when they are most needed [3]. These studies have shown that decision policies can be learned from behavioral data to time interventions for greater effect. Crucially, safety remains a paramount concern. For instance, Weimann and Gißke [4] provide a recent review of RL-driven behavioral DTx, noting that although some implementations include simple safety measures (eg, time caps on intervention delivery), they lack the type of feedback prediction and constraint-aware planning architecture we propose. Researchers emphasize that in health care, RL policies should generally be developed or validated on existing datasets (offline learning) or realistic simulations, rather than through trial and error on patients, to avoid unsafe experimentation. Such recommendations align with safe RL techniques discussed by Jayaraman et al [3] and García and Fernández [5], who advocate approaches such as offline training and conservative Q-learning to improve safety; however, these methods have yet to be integrated into real-time DTx personalization. For example, an offline RL approach for sepsis treatment used a conservative algorithm that penalized actions not well supported by the data, thereby avoiding extreme or high-risk recommendations [6]. This illustrates that RL can be adapted to prioritize patient safety by incorporating constraints and risk awareness into the learning process. Beyond RL, simulation-based optimization has also shown promise in digital health. For instance, Killian et al [7] recently optimized a digital health intervention to improve patient engagement while managing risks, underscoring the value of in silico experimentation for safe DTx personalization.

### Intermediate Feedback Signals

Using intermediate feedback signals to guide therapy decisions is a well-established concept in medicine. In drug development, for instance, surrogate end points (biomarkers or short-term measures that correlate with true clinical outcomes) are often used to evaluate treatments more quickly when final outcomes are difficult to obtain. Similarly, DTx can monitor proxy measures of patient progress during treatment. Many current DTx already collect rich in-treatment data as feedback. For example, a Food and Drug Administration (FDA)–cleared video game–based therapy for pediatric ADHD (AKL-T01) automatically adapts its gameplay difficulty based on the child’s performance and attention metrics, and those intermediate metrics correlate with longer-term clinical improvements in attention [8,9].

Likewise, in mental health DTx, momentary self-reported mood or stress ratings are often used to personalize intervention timing [3]. Machine learning models have also shown success in predicting short-term mood fluctuations using passive data from smartphones and wearables [10,11], suggesting that a DTx could forecast a patient’s immediate response to an intervention. Engagement and adherence are another critical class of feedback signals: many behavioral interventions rely on the patient’s active participation, so intermediate measures such as app use time, task completion rate, or session attendance are strong indicators of whether the treatment is on track. In summary, prior work indicates that short-term feedback signals (performance metrics, momentary symptoms, and engagement levels) can serve as effective surrogates for long-term outcomes in DTx. Although existing DTx often track these signals passively or use them for simple rule-based triggers, SAFE_DTx distinctly leverages them as the primary optimization objective within a constraint-aware planning loop, enabling proactive, data-efficient therapy adaptation.

### Safety and Explainability in AI-Driven DTx

The use of AI in health care, and in DTx in particular, has prompted substantial discussion about ensuring safety, transparency, and accountability. Researchers have highlighted risks such as algorithmic bias, lack of interpretability, and unpredictable behavior in clinical AI systems [2]. In response, there is increasing emphasis on making AI explainable and demonstrably safe. For DTx, this means that the AI-driven decision logic should be transparent to clinicians and patients and that the system should have safeguards to prevent harm. Several frameworks and guidelines have emerged to manage these risks. For example, Denecke et al [12] propose a DTx Risk Assessment Canvas to systematically identify potential safety risks in DTx, although this framework does not address the unique challenges of AI-driven or real-time adaptive DTx. Regulatory agencies such as the US FDA and the European Commission are developing oversight approaches for AI-based medical software, including software as a medical device (SaMD). These approaches emphasize good machine learning practices (eg, training on representative data, prospective validation, and monitoring for performance drift) [2]. The FDA’s proposed action plan for AI-ML–based SaMD outlines a risk-based approach to approval and highlights the need for algorithm transparency [13]. For example, regulators classify an AI-driven intervention’s risk level partly by the role it plays in care; an assistive, constrained decision support tool may be viewed as lower risk than a fully autonomous system [14]. This has practical implications for DTx developers: designing the AI to make only incremental, bounded recommendations may align with current regulatory frameworks prioritizing safety oversight. In summary, current regulatory and ethical trends support the type of safe-by-design approach embodied by SAFE_DTx, wherein AI personalization is kept within strict safety and explainability boundaries. Building on these foundations, SAFE_DTx addresses clinical safety concerns through a 2-module architecture that separates feedback prediction from treatment planning, enabling transparent decision-making with explicit safety constraints.

## SAFE_DTx Framework

Building on these foundational concepts, we present SAFE_DTx as a modular closed-loop system designed to enable safe AI-driven personalization in DTx. As illustrated in Figure 1, the architecture comprises 2 primary modules working in tandem with a continuous monitoring feedback loop.

**Figure 1:**
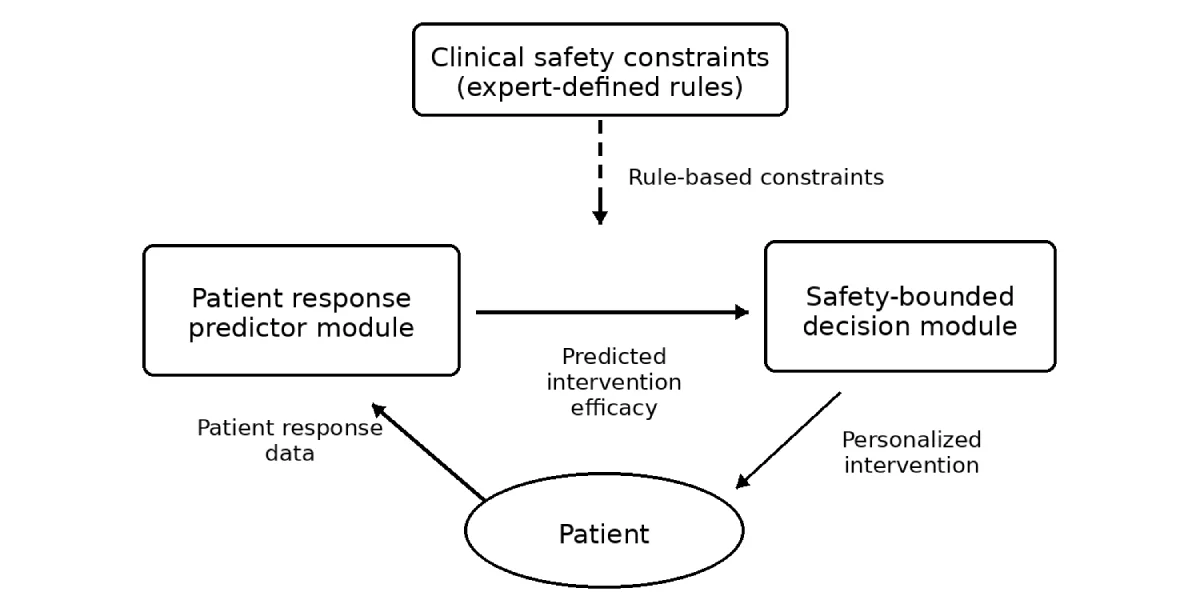
Two-step closed-loop architecture of SAFE_DTx. The patient response predictor module outputs predicted intervention efficacy, while the safety-bounded decision module applies strict rule-based constraints before personalized intervention selection.

### AI Feedback Prediction Module

This module is responsible for modeling the patient’s short-term response dynamics. It ingests diverse data about the patient’s current state, including recent interactions with the DTx (which modules were completed and how the patient performed), recent feedback signals (eg, engagement metrics, symptom self-reports, and physiological readings), and contextual information such as the time of day or patient history. Using this information, the AI module predicts the expected feedback outcome for each candidate next action. For example, it might estimate “if the patient is given a relaxation exercise now, their stress level one hour later will decrease by 2 points on a 0–10 scale, with 90% confidence.” Such predictions could be generated by machine learning models trained on historical data from similar patients—for instance, a recurrent neural network [15] that captures how the patient’s state evolves with each type of intervention. Crucially, the AI module should also provide an uncertainty measure with each prediction (eg, a CI or probability distribution over possible outcomes). Well-calibrated uncertainty quantification is important for safety [6,13]. Practically, algorithmic uncertainty bounds must be translated into interpretable clinical confidence scores. If this score falls below a predefined safety threshold, the planner automatically defaults to a clinically validated, lowest-risk fallback protocol, while flagging the patient’s state on a clinician dashboard for actionable human review. Techniques such as Bayesian neural networks or ensemble models (which aggregate multiple models or use probabilistic methods to quantify uncertainty) can be used to obtain these confidence estimates. The AI model would be updated periodically (retrained or fine-tuned) as more data from the specific patient become available, but such updates must be performed cautiously and offline to avoid sudden, untested shifts in behavior during active treatment.

### Safe Planning Module

This module takes the predictive model’s outputs (the estimated outcome for each potential action, plus associated uncertainty) and formulates an optimization problem to choose the next action (or sequence of actions) that maximizes the expected intermediate feedback signals while satisfying all safety constraints. If using an RL-based approach, the planner could simulate different policy options using the model and evaluate their expected long-term reward and then choose the best action according to the learned policy. If using an optimization approach, the planner strictly subordinates its primary objective (eg, maximizing predicted intermediate feedback) to deterministic clinical boundaries. This architectural hierarchy ensures that the solver automatically filters out any action violating expert-defined safety constraints before calculating the optimal intervention schedule. In real time, as noted, the planning may often be simplified to a 1-step lookahead where the system selects the action with the highest predicted immediate improvement that satisfies all safety constraints. However, the architecture also supports a longer planning horizon if needed—for example, simulating a sequence of daily actions to plan an entire week’s therapy in advance, if the model can project that far. Crucially, safety constraints must be explicitly defined per clinical domain by medical experts prior to deployment. Rather than adapting to condition-specific clinical nuances during runtime, the planning module treats all expert-defined rules uniformly as strict mathematical boundary conditions. This architectural separation allows the system to systematically filter out violating actions before evaluating predicted rewards, regardless of the underlying clinical context. For instance, if the patient’s recent feedback indicates significant fatigue or distress, the planner might be forbidden from choosing a high-intensity exercise next. By incorporating such rules, the planner ensures that any recommended treatment plan stays within clinically acceptable thresholds for safety and tolerability.

### Closed-Loop Delivery and Monitoring

Once the safe planning module selects an optimal action, that intervention is delivered to the patient via the DTx platform. The patient’s response to the intervention (eg, whether they completed the task, their symptom rating afterward, or any adverse signs) is then observed and fed back into the system. This closes the loop: the new feedback is logged by the AI prediction module, updating the patient’s state. The next decision cycle then repeats with the updated state information. This continuous monitoring allows SAFE_DTx to dynamically adjust the intervention and also to catch any warning signs. If at any point the feedback indicates danger (eg, a patient’s mood drops below a predefined safety threshold or they report suicidal ideation), SAFE_DTx can be configured with an immediate safety override. In such a scenario, the planner’s sole “action” would be to escalate care—for example, by triggering an alert to a clinician or caregiver—rather than attempting a digital intervention in isolation. Thus, the system not only optimizes therapy but also acts as a safety net, reverting to human intervention when necessary.

Overall, the 2-module architecture of SAFE_DTx (with separate prediction and planning components) provides clarity and flexibility. Each module can be validated independently, and explicit safeguards (eg, uncertainty-aware defaults or hard limits) can be inserted at multiple points in the decision process. This modular design means that clinicians and regulators can review and evaluate the planner’s decision rules and the predictor’s accuracy separately. Our design deliberately integrates modern machine learning—to predict a patient’s short-term response—with rigorous operational constraints to ensure that each chosen action is safe and clinically sound. It is specifically aimed at addressing key challenges in AI-driven health care, namely, data scarcity; the need for transparency; and, above all, patient safety. These design differences are summarized in Table 1, which compares SAFE_DTx and conventional AI personalization approaches across 4 key dimensions: optimization target, safety mechanism, data dependency, and transparency.

**Table 1:** Key differences between SAFE_DTx and conventional AI personalization approaches.

Characteristics	SAFE_DTx	Conventional AI personalization
Primary optimization target	Intermediate, clinically relevant feedback signals (eg, engagement and daily symptoms)	Often distant, long-term health outcomes (eg, remission)
Safety mechanism	Explicit safety constraints in a dedicated planning module, uncertainty-aware predictions, and human escalation protocols	Often relies on model robustness or implicitly learned safety from data, with a risk of unsafe exploration
Data dependency for optimization	Leverages richer, real-time intermediate data for adaptation	Requires scarce, delayed long-term outcome data, limiting adaptability
Transparency and validation	Modular architecture with prediction and planning separation; planning module rules are scrutable and clinically verifiable	Often relies on “black-box” systems, with decision logic that is difficult to interpret or validate independently

## Illustrative Simulation

To evaluate the architectural feasibility of SAFE_DTx, we conducted a proof-of-concept simulation in a controlled in silico environment. It must be explicitly noted that these simulation results are strictly illustrative and do not constitute evidence of clinical effectiveness. We created a simulated patient population to emulate how patients might respond to various DTx personalization strategies. For demonstration, we simulated a cohort of 100 virtual patients with depression over a 56-day intervention period, modeling daily mood and fatigue as dynamic state variables. The simulated patient population was modeled not as perfectly rational actors but as entities subject to stochastic behavioral changes to represent erratic engagement. This environment serves specifically as a computational stress-test sandbox to verify the framework’s constraint enforcement mechanisms. It is designed to illustrate architectural logic, rather than to replicate complex human psychology or demonstrate clinical efficacy. We also incorporated occasional adverse events in the simulation: for example, if interventions were too frequent or intense, there was a small probability of a sharp mood drop to represent a negative outcome. The immediate reward at each step was defined as the patient’s engagement with the therapy (ie, whether they completed that day’s intervention), which our SAFE_DTx agent aimed to maximize as an intermediate feedback signal.

Within this simulated environment, we compared our safe optimization policy against two baseline strategies. First, a greedy policy (unsafe baseline); this is a naive personalization strategy that tries to maximize the final outcome without using intermediate feedback. In practice, this greedy strategy always selected the action that historically yielded the best long-term result, regardless of the patient’s current state or short-term feedback. It did not incorporate any safety constraints. Second, a static protocol (no personalization); this is a standard fixed treatment schedule with no AI-driven adaptation. This represents current practice in which every patient receives the same sequence of interventions on a set schedule.

By running many simulation trials (50 independent runs per strategy), we measured several outcomes to evaluate each strategy. We tracked the cumulative intermediate feedback signals (total engagement over the 56 days, which the SAFE_DTx agent explicitly tried to maximize), as well as the final clinical outcome for each strategy (to confirm that improving short-term feedback indeed translates into better long-term results), and any safety violations (instances where a simulated patient’s state crossed an unsafe threshold, representing a potential adverse event or clinically unacceptable deterioration).

Our results showed that SAFE_DTx outperformed both baselines on all key metrics. The safe optimization agent achieved greater total engagement (intermediate feedback signals) over the course of therapy and also led to better final outcomes on average. Notably, optimizing the feedback signals yielded superior final outcomes within the simulation’s predefined state transition logic: by the end of 56 days, the SAFE_DTx group had a higher mean outcome than both the static and greedy strategy groups. Although this confirms the algorithm’s computational capability to effectively optimize proximal targets under constraints, the true clinical correlation between these specific intermediate signals and long-term outcomes remains a subject for future empirical validation. Furthermore, SAFE_DTx strictly avoided safety breaches. Across simulations, the SAFE_DTx policy incurred 0 instances of violating the predefined safety thresholds, whereas the unconstrained greedy policy led to multiple such violations (eg, triggering simulated adverse events in more than 50% of the runs). The static protocol, while safe by virtue of being conservative, was less effective, achieving lower engagement and smaller improvements in outcomes.

Figure 2 illustrates 1 aspect of these findings: the average mood trajectory of patients under each strategy. The SAFE_DTx policy maintained a higher and more stable average mood over time, reflecting improved emotional stability under safety-aware optimization, whereas the greedy strategy achieved rapid initial gains in mood but then suffered severe crashes in the later weeks (demonstrating the risks of an aggressive approach without safety limits). The static schedule produced only modest initial improvement and then plateaued, as it did not adapt to the patient’s needs. Table 2 summarizes the overall comparison: SAFE_DTx achieved the highest engagement (89% adherence rate) and the lowest mood volatility (most stable mood), and it had 0 “unsafe days,” in contrast to the greedy strategy, which led to safety incidents on more than half of the simulated treatment days. The static strategy fell in between, with moderate engagement and no explicit safety incidents but also less overall improvement.

These in silico findings illustrate the architectural plausibility of the framework. Although they do not constitute empirical clinical proof, the results logically demonstrate that optimizing short-term feedback signals within structurally separated safety boundaries can systematically prevent constraint violations in a simulated environment. The current evaluation is strictly constrained to a stress-test sandbox. It relies on stochastic behavioral proxies rather than complex human psychology and uses heuristic baseline comparators solely to illustrate the extreme boundaries of unconstrained adaptation vs nonadaptive rigidity, rather than benchmarking against advanced safe RL algorithms (eg, constrained policy optimization). Therefore, transitioning from this architectural proof of concept to clinical application mandates future studies incorporating formal algorithmic benchmarking and closed-loop empirical validation. In this way, the simulation served as a valuable sandbox to refine SAFE_DTx prior to clinical deployment. By demonstrating in silico that feedback-driven optimization can consistently improve intermediate and final outcomes without safety breaches, we build confidence that the framework is fundamentally sound and ready for cautious real-world testing.

**Figure 2:**
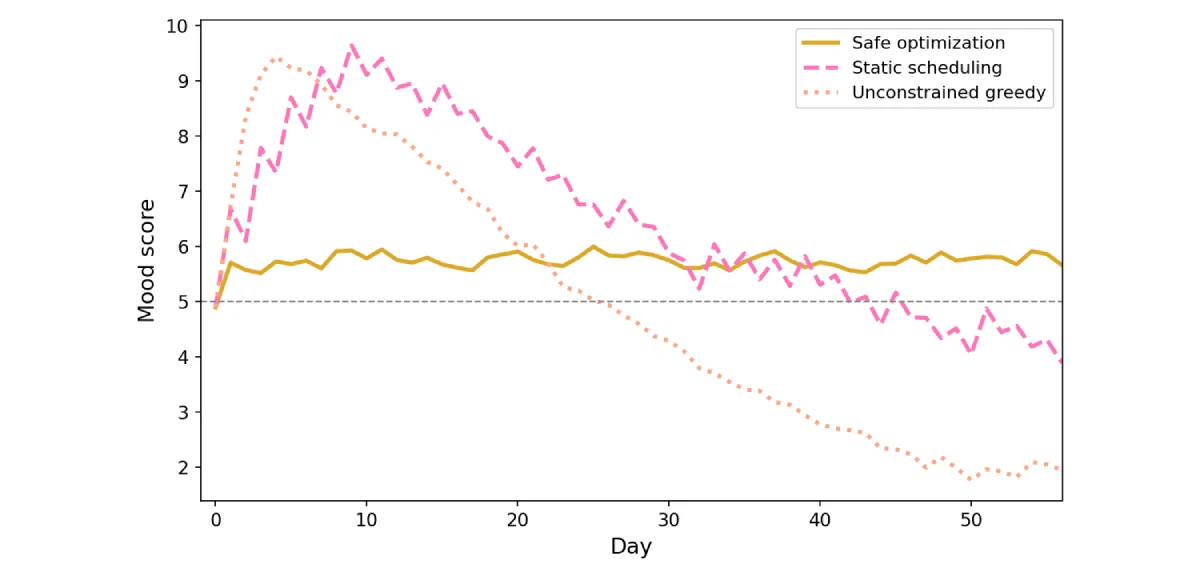
Average mood trajectory over 56 days across treatment strategies. The graph shows the mean daily mood score of 100 simulated patients under 3 different strategies. The safe optimization strategy (solid yellow line) maintained a stable mood level over time, reflecting emotional stability under safety-aware optimization. The static scheduling strategy (dashed pink line) showed initial mood improvement but gradually declined due to unadapted scheduling. The unconstrained greedy strategy (dotted orange line) achieved sharp early gains but experienced severe mood crashes in the second half, illustrating risks of overintensification without safety constraints.

**Table 2:** Simulation results across strategies^a^.

Strategies	Engagement rate, n (%)	Mood volatility, mean (SD)	Unsafe days, n (%)
Safe optimization	1146 (88.8)	0.93 (0.09)	0 (0.0)
Unconstrained greedy	2098 (41.1)	3.04 (0.73)	2895 (51.7)
Static scheduling	2034 (72.6)	2.56 (0.82)	1227 (21.9)

^a^The table summarizes the outcomes of a 56-day simulation involving 100 virtual patients with depression under 3 treatment strategies: safe optimization, unconstrained greedy, and static scheduling.

## Discussion

### Illustrative Applications in Different Therapeutic Areas

Although our simulation focused on a depression scenario, the SAFE_DTx framework is general and can be applied to various conditions where DTx are used. Here, we consider 2 examples to illustrate how SAFE_DTx might operate in different therapeutic domains.

DTx for depression range from stand-alone mobile apps delivering cognitive behavioral therapy (CBT) exercises to platforms that combine behavioral activation, mood tracking, and coaching. In depression, mood and anxiety levels can fluctuate daily, and engagement with therapy is often erratic due to motivational difficulties. SAFE_DTx could enable a form of JITAI for depression management, adjusting support based on the patient’s momentary state. The AI predictor would forecast the patient’s near-term mood or stress level using recent inputs, including self-reported mood ratings and passive smartphone data such as activity or social interaction patterns [10,11]. The planner then chooses an intervention intended to improve the projected mood or prevent a decline.

Safety is paramount in depression treatment, especially concerning suicidal ideation. In a SAFE_DTx implementation for depression, the system would be configured with strict safety rules related to any signs of acute risk. For instance, if any feedback suggests emergent suicidality or a severe spike in hopelessness, the SAFE_DTx planner would not attempt any digital intervention on its own. Instead, its sole “action” in that situation would be to immediately escalate care—for example, by triggering an alert to a human clinician or caregiver for urgent evaluation—thereby ensuring that the patient receives appropriate human-led care when needed. This approach ensures that autonomous personalization never oversteps its bounds in high-risk scenarios; the AI remains a supportive tool and hands control back to clinicians when serious risks arise.

The illustrative application in depression demonstrates how SAFE_DTx can be tailored to specific conditions. The framework leverages condition-specific feedback signals and interventions, while the core principle remains the same: use AI predictions to personalize the treatment in real time and strictly enforce safety through constraints and human oversight. As DTx expand to other areas (eg, diabetes management, anxiety disorders, and cognitive rehabilitation), similar safe personalization strategies could be devised. The generality of SAFE_DTx suggests that it could serve as a blueprint for next-generation DTx that are both adaptive and trustworthy.

### Limitations

#### Need for Clinical Validation

As emphasized, the current evaluation is strictly illustrative and does not serve as empirical evidence of clinical effectiveness. Transitioning from this structural proof of concept to real-world clinical application requires a phased validation pathway: (1) offline validation using historical DTx log data, (2) an open-loop pilot generating recommendations for clinician review, and (3) a closed-loop RCT to confirm noninferiority in safety and superiority in engagement. Factors that were simplified or absent in our simulations—such as the placebo effect, variations in patient adherence, comorbidities, and unpredictable human behaviors—could influence outcomes. Thus, there is uncertainty about how well improvements in intermediate feedback will translate to long-term clinical benefits in live use, and only empirical testing can bridge that gap. Gradual, carefully monitored deployment (eg, using SAFE_DTx in an observational study or alongside a clinician who can veto actions) might be a prudent path to building trust and evidence incrementally.

#### Regulatory and Liability Considerations

Beyond technical performance, there are practical considerations regarding clinical responsibility and regulatory approval. Although broad legal allocation of liability in SaMD remains complex, SAFE_DTx addresses this technically. If deployed strictly as a “decision support” tool with human-in-the-loop oversight, liability largely remains with the prescribing clinician. Furthermore, by architecturally isolating the explicit setting of constraints by clinical panels prior to deployment, the framework provides an engineering safeguard that enhances technical traceability, which may assist stakeholders in clarifying system behaviors during liability assessments. Current regulatory frameworks do not yet fully address such scenarios. Clear guidelines, and possibly new regulatory categories, may be needed to clarify liability in the context of adaptive AI interventions. Those developing and deploying SAFE_DTx should work closely with regulators from the earliest stages. The FDA’s evolving guidance on adaptive algorithms and the European Union’s proposed Artificial Intelligence Act [14] will likely shape the requirements for a system such as SAFE_DTx. Positioning the framework as a decision support tool (as opposed to an autonomous therapy) may align better with existing regulatory pathways for lower-risk medical software. Additionally, educating clinicians about how the system works will be important for real-world integration. Clinicians will need to understand the rationale behind SAFE_DTx’s recommendations, its limitations, and when it may be necessary to override or intervene. Building this human-AI partnership in care delivery is a social challenge that goes hand in hand with the technical solution.

#### Theoretical Guarantees vs Empirical Safety

Our framework currently enforces safety via design constraints and has shown no safety violations in testing, but we have not formally *proven* that the system will never take an unsafe action under all possible conditions. In other words, there is not yet a mathematical guarantee of safety. Providing formal safety assurances (for instance, using verification methods or proving certain properties about the algorithm) would further strengthen confidence, especially for high-stakes medical use. This remains an area for future work.

### Future Directions

#### Future Research Directions

Despite these limitations, the potential of safe optimization in DTx is significant. We outline a few directions for future research to strengthen and expand SAFE_DTx. Each of these directions represents an opportunity to make SAFE_DTx more robust, generalizable, and usable. Ultimately, achieving the full vision of SAFE_DTx will require interdisciplinary collaboration—engaging experts in AI, human-computer interaction, clinical practice, and health policy.

#### Hybrid Modeling Approaches

Combining data-driven learning with knowledge-based rules or physiological models could further strengthen the framework. For instance, the planner’s safety constraints or initial policy could be informed by established clinical guidelines (eg, maximum exercise heart rate rules in a cardiac rehabilitation DTx), and then the AI can fine-tune the policy within those bounds. This hybrid approach might accelerate learning while maintaining safety and interpretability.

#### Incorporating Additional Data Modalities

Richer data sources such as genomics, biomarkers, or medication data could be integrated into the personalization loop. For example, if a DTx is used alongside pharmacotherapy, SAFE_DTx could adjust its recommendations on days when medication is taken (or based on predicted drug side effects) to better synergize with pharmacokinetics. Multimodal data could improve the predictor’s accuracy and open up new personalization opportunities, albeit with the requirement to also incorporate any modality-specific safety checks.

#### Expanded Simulation Studies

Before moving to large clinical trials, it would be beneficial to create more comprehensive simulation environments or “digital twin” models for other conditions to stress-test SAFE_DTx. For instance, one could simulate a cohort of patients with diabetes using a DTx for blood sugar management to ensure SAFE_DTx handles rare events such as hypoglycemia correctly. Similarly, simulations for anxiety disorders or rehabilitation therapies could reveal condition-specific challenges. Such extensive in silico experimentation can uncover potential safety issues and edge cases in a risk-free manner, allowing the framework to be refined further.

#### Integration With Clinical Workflow

Research should explore how to effectively integrate SAFE_DTx into the routine clinical workflow. The system might provide periodic summary reports to human clinicians, for example: “The patient’s digital therapy data indicate consistently high stress on Mondays; consider adjusting Monday’s in-person therapy or workload.” By communicating salient insights and remaining under human oversight, SAFE_DTx can augment care without supplanting it. Designing user interfaces and alert mechanisms that deliver the right information at the right time to clinicians (and feedback to patients to maintain their engagement and trust) will be key for real-world adoption.

### Conclusions

In this paper, we introduced SAFE_DTx, a framework that combines AI-driven feedback prediction with safe planning to personalize digital therapy. Unlike conventional approaches, SAFE_DTx explicitly optimizes intermediate feedback signals under medical safety constraints, enabling fine-grained adaptation to the patient while proactively managing risk. By shifting the optimization target to intermediate feedback signals, the framework mitigates the data scarcity associated with distant clinical outcomes. Concurrently, by decoupling predictive mechanisms from deterministic constraints, the architecture helps address AI opacity and ensures patient safety. Our illustrative examples in mental health and other domains demonstrate how SAFE_DTx adapts to different contexts by optimizing short-term feedback. Although these simulation-based observations serve to validate the structural logic rather than provide clinical proof, they establish a plausible foundation for the use of this modular approach to proactively manage patient risk.

Importantly, although SAFE_DTx has so far been demonstrated only in simulation (and not yet tested in clinical settings), it offers a concrete methodology for moving forward. It suggests what types of data to collect, how to design trials focusing on intermediate outcomes, and how to gradually build trust in an AI-driven system through simulations and phased deployments. As DTx continue to evolve, we envision that such safe optimization systems could become a core part of next-generation “intelligent” DTx products. These would not replace clinicians but rather extend their reach, ensuring that each patient’s digital treatment is optimized in real time according to the best evidence and individual response, all while staying within clearly defined safety guardrails informed by medical science.

The integration of AI and optimization in DTx offers a promising pathway to deliver personalized, adaptive treatment at scale. SAFE_DTx provides a foundation for doing so in a way that meets the high safety and reliability standards of health care. We hope this work stimulates further research and development to refine the SAFE_DTx approach, gather clinical evidence, and ultimately translate it into real-world interventions that improve patient outcomes. The journey from conceptual framework to clinical reality will require close collaboration between data scientists, clinicians, regulatory experts, and patients, but the potential rewards—in terms of more effective and safer therapies—make this a worthwhile endeavor.

## Abbreviations

ADHD: attention-deficit/hyperactivity disorder
CBT: cognitive behavioral therapy
DTx: digital therapeutics
FDA: Food and Drug Administration
JITAI: just-in-time adaptive intervention
RL: reinforcement learning
SaMD: software as a medical device

## Data Availability

No real-world patient data were used. The simulation code and parameters are available from the author upon reasonable request.

## References

[1] Wang C, Lee C, Shin H (2023). Digital therapeutics from bench to bedside. NPJ Digit Med.

[2] Challen R, Denny J, Pitt M, Gompels L, Edwards T, Tsaneva-Atanasova K (2019). Artificial intelligence, bias and clinical safety. BMJ Qual Saf.

[3] Jayaraman P, Desman J, Sabounchi M, Nadkarni GN, Sakhuja A (2024). A primer on reinforcement learning in medicine for clinicians. NPJ Digit Med.

[4] Weimann TG, Gißke C (2024). Unleashing the potential of reinforcement learning for personalizing behavioral transformations with digital therapeutics: a systematic literature review. Proceedings of the 17th International Joint Conference on Biomedical Engineering Systems and Technologies - HEALTHINF.

[5] García J, Fernández F (2015). A comprehensive survey on safe reinforcement learning. J Mach Learn Res.

[6] Tu R, Luo Z, Pan C, Wang Z, Su J, Zhang Y, Wang Y (2025). Offline safe reinforcement learning for sepsis treatment: tackling variable-length episodes with sparse rewards. Hum Cent Intell Syst.

[7] Killian JA, Jain M, Jia Y, Amar J, Huang E, Tambe M (2024). New approach to equitable intervention planning to improve engagement and outcomes in a digital health program: simulation study. JMIR Diabetes.

[8] Kollins SH, DeLoss DJ, Cañadas E, Lutz J, Findling RL, Keefe RS, Epstein JN, Cutler AJ, Faraone SV (2020). A novel digital intervention for actively reducing severity of paediatric ADHD (STARS-ADHD): a randomised controlled trial. Lancet Digit Health.

[9] Stamatis CA, Heusser AC, Simon TJ, Ala'ilima T, Kollins SH (2024). Real-time cognitive performance metrics derived from a digital therapeutic for inattention predict ADHD-related clinical outcomes: replication across three independent trials of AKL-T01. Transl Psychiatry.

[10] Pratap A, Atkins DC, Renn BN, Tanana MJ, Mooney SD, Anguera JA, Areán PA (2019). The accuracy of passive phone sensors in predicting daily mood. Depress Anxiety.

[11] Islam R, Zhang T, Bae SW (2024). Moodcam: mood prediction through smartphone-based facial affect analysis in real-world settings. 2024 IEEE Smart World Congress (SWC).

[12] Denecke K, May R, Gabarron E, Lopez-Campos GH (2023). Assessing the potential risks of digital therapeutics (DTX): the DTX Risk Assessment Canvas. J Pers Med.

[13] (2021). Artificial intelligence/machine learning (AI/ML)-based Software as a Medical Device (SaMD) action plan. U.S. Food & Drug Administration.

[14] Vokinger KN, Gasser U (2021). Regulating AI in medicine in the United States and Europe. Nat Mach Intell.

[15] Choi E, Bahadori MT, Kulas JA, Schuetz A, Stewart WF, Sun J, Lee DD, von Luxburg U, Garnett R, Sugiyama M, Guyon I (2016). RETAIN: an interpretable predictive model for healthcare using reverse time attention mechanism. NIPS'16: Proceedings of the 30th International Conference on Neural Information Processing Systems.

